# Elevated KIR expression and diminished intensity of CD7 on NK cell subsets among treatment naïve HIV infected Ethiopians

**DOI:** 10.1038/s41598-022-18413-3

**Published:** 2022-08-30

**Authors:** Henok Andualem, Mulualem Lemma, Amare Keflie, Meseret Workeneh, Birhanu Ayelign, Yayehyirad Tassachew, Lidya Hailu, Alene Geteneh, Adane Mihret, Martha Zewdie, Rawleigh Howe

**Affiliations:** 1 Department of Medical Laboratory Science, College of Medicine and Health Sciences, Debre Tabor University, P.O. Box 272, Debre Tabor, Ethiopia; 2 Department of Immunology and Molecular Biology, School of Medical Laboratory Science, College of Medicine and Health Sciences, University of Gondar, Gondar, Ethiopia; 3 School of Medical Laboratory Sciences, College of Medicine and Health Sciences, Hawassa University, Hawassa, Ethiopia; 4 Department of Chemistry, College of Natural and Computational Science, Debre Tabor University, Debre Tabor, Ethiopia; 5 Department of Medical Laboratory Science, College of Health Sciences, Woldia University, Woldia, Ethiopia; 6 Armauer Hansen Research Institute, Addis Ababa, Ethiopia

**Keywords:** Immunology, Microbiology, Infectious diseases

## Abstract

Natural killer (NK) cells are crucial effector cells of the innate immune response to viral infections, including HIV, through cytolytic activity and the production of cytokines with anti-HIV activities. We recruited 15 treatment naïve HIV patients and 16 healthy controls (HC) to assess NK cell subsets or expression of multiple markers by flow cytometry. The frequency of circulating CD56^bright^CD16^−ve^ and CD56^dim^CD16^bright^ NK cell subsets was significantly lower among the HIV group than in HC. The CD56^−ve^CD16^bright^ subset was higher in HIV patients, but this was only apparent when gated among total NK cells, not total lymphocytes. NK cells among HIV participants also showed a lower and higher frequency of CD8 and HLA-DR expressing cells, respectively. In addition, CD7 median fluorescent intensity and CD2^+^CD7^−^ frequencies were significantly lower in HIV patients. A distinct population of KIR3DL1/S1 cells was unexpectedly higher among CD56^bright^CD16^−ve^ NK cells in HIV patients. In conclusion, this study in the Ethiopian setting confirms many previous findings, but the down-regulation of CD7 and enhanced KIR3DL1/S1 within the CD56^bright^ subsets have not been widely reported among HIV patients and merit further research.

## Introduction

An effective immune response towards invading microbes requires the combined action of innate and adaptive immunity. Natural killer (NK) cells are effector lymphocytes of the innate immune response, and lyse cells infected with the virus, including human immunodeficiency virus (HIV)^1^. They also participate in determining the fate of adaptive immune responses through secretion of cytokines, such as interferon γ (IFN- γ)^2^. NK cell function is primarily determined by germline-encoded activating and inhibitory receptors, which after integration results in NK cell activation or not^3^. NK cells are typically identified as CD3^−^CD56^+^ cells. They can be further classified based on the expression of CD16^4^. The majority of peripheral blood NK cells are CD56^dim^CD16^bright^ and usually cytotoxic^5^, whereas most of the remaining are CD56^bright^CD16^−ve^ and produce more cytokines and chemokines than the former^6^. The CD56^bright^ subsets are highly proliferating, and considered immature precursor subsets for CD56^dim^ cells. The CD56^dim^ has been proposed to undergo further irreversible differentiation, progressively losing immature inhibitory NK cell receptors, such as natural killer group 2 A (NKG2A), and begin the sequential acquisition of killer like immunoglobulin receptors (KIRs) and CD57^7,8^.

Numerous studies have shown that HIV infection alters subsets distribution and the expression of receptors on NK cells. This includes the enhanced appearance of a functionally impaired subset, the normally infrequent CD56^−ve^CD16^bright^ subset, and the decrease in frequencies of the CD56^bright^CD16^−^ and CD56^dim^CD16^bright^ subsets^6,9–11^. HIV also upregulates inhibitory while downregulating activation receptors that could support disease progression^12,13^. Although highly active anti-retroviral therapy (HAART) restores most NK cell functions and normalizes subset distribution, incomplete recovery of subsets has been reported even after long-term HAART^14^. Incomplete recovery may, in turn, affect the recovery of adaptive immune cell functions, including the chronic alterations of T cells and associated immune dysfunction^15^.

The expression of certain markers, such as CD8 and KIR3DL1/S1 on NK cells is associated with slow HIV progression to AIDS^16,17^. A higher frequency of CD57^+^ NK cells has also been shown to be associated with better CD4 recovery and low HIV viral load^18^. Furthermore, activated NK cells expressing HLA-DR among HIV patients may be related to delayed recovery of CD4 count after HAART^19^. Adaptive or memory-like functions or phenotypes in human cytomegalovirus (HCMV) infections have been identified among NKG2C^+^ NK cells. Hence, NK cell phenotypes may reveal valuable information about disease prognosis, particularly among HAART naïve individuals.

While the global number of new HIV infections is declining, southern and eastern African regions comprise 47% of the global infections^20^. The prevalence of HIV infection among individuals aged 15–49 in Ethiopia is 0.9%^21^. Few comprehensive studies evaluating NK phenotype have been done in sub-Saharan Africa (SSA), and none to the best of our knowledge in Ethiopia. Given that genetic and environmental factors impacting disease may differ in these settings, our objective in the current study was to assess NK phenotypes of HIV patients and controls, and determine whether or not such findings were consistent or not with prior studies.

## Results

### Characteristics of the study participants

A total of 31 study participants (15 HAART naïve HIV patients and 16 healthy controls) were enrolled. The majority were female, comprising 9/15 (60%) in the HIV group and 10/16 (62.5%) in healthy controls (HC) groups. The median age of HIV and HC groups was 32 (range 20–46) and 28 (range 19–42), respectively (Table 1). The median viral load in HAART naïve HIV patients was 102,566.5 copies/ml (range 4039–257,615). All HIV patients had the disease for greater than 6 months.

**Table 1:** Characteristics of HAART naïve HIV patients and healthy controls.

Characteristics	HIV patients	Healthy controls
Case	15	16
Sex (male/female)	6/9	6/10
Age (median with range)	32 (20–46)	28 (19–43)
Residence (urban/rural)	14/1	12/4
HIV viral load level (median with range)	102,566.5 copies/ml (4039–257,615)	N/A
Duration of infection at the time of diagnosis	> 6 months	N/A

### NK cells in HIV patients and healthy controls

**Figure 1:**
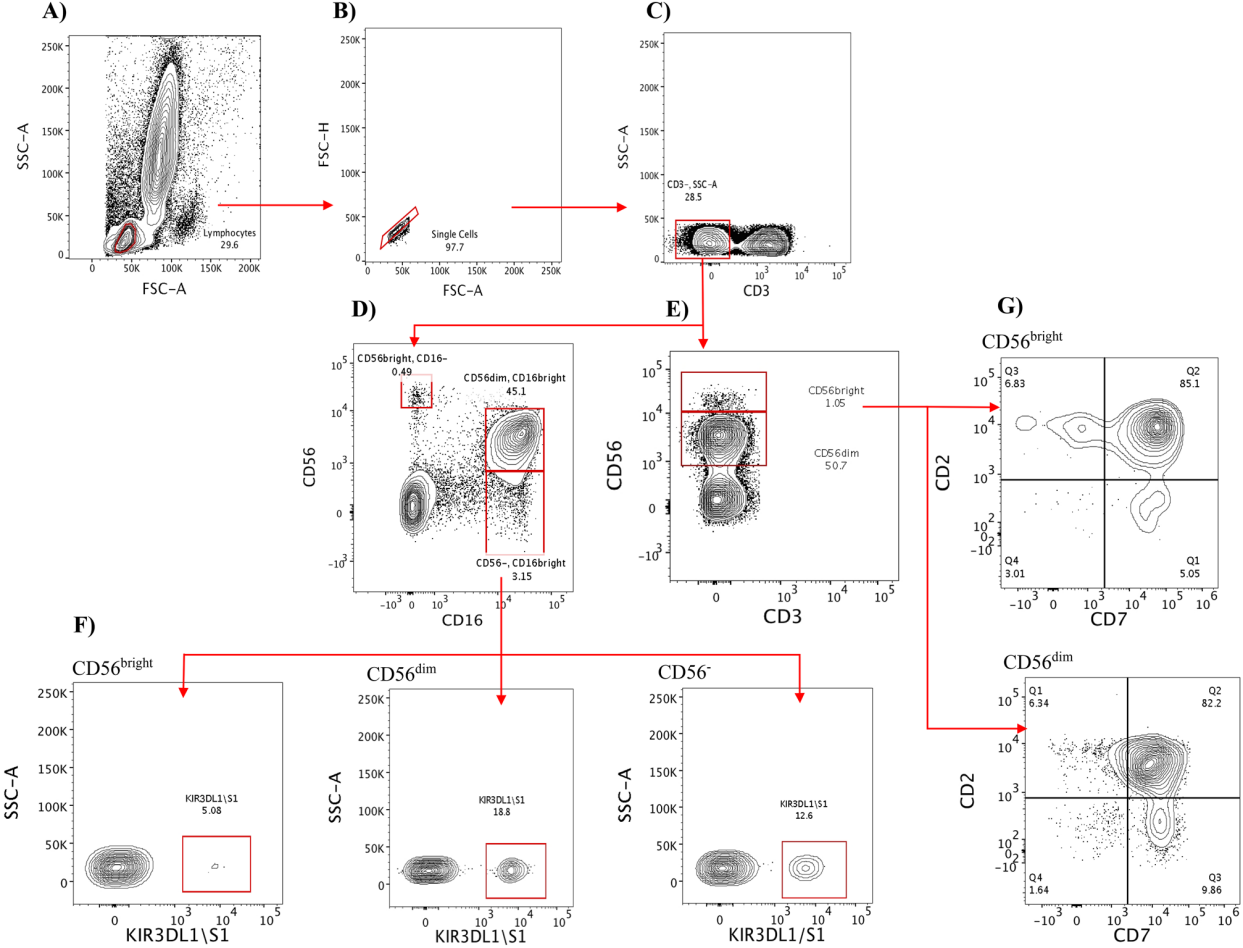
Gating strategy for NK cell subsets. (**A**) The lymphocytes were first identified based on forward (FSC) and side scatter (SSC) analysis; doublets were subsequently excluded with a FSC-H vs FSC-A plot (**B**). (**C**) CD3^−^ cells were then gated, followed by the definition of the three NK cell subsets in the panels containing CD56 and CD16 (**D**), or two subsets in the panels without CD56 antibody alone (**E**). (**F**) The three subsets (CD56^bright^CD16^−ve^ in the lower-left corner, CD56^dim^CD16^bright^ in the middle, and CD56^−ve^CD16^bright^ in the lower right corner) were further examined for CD8 and KIR3DL1/S1 expression. (**G**) The expression of CD2 and CD7 among CD56^bright^CD16^−ve^ (top right corner) and CD56^dim^CD16^bright^ (lower right corner) is shown**.** The representative sample for gating illustration was taken from healthy controls.

**Figure 2:**
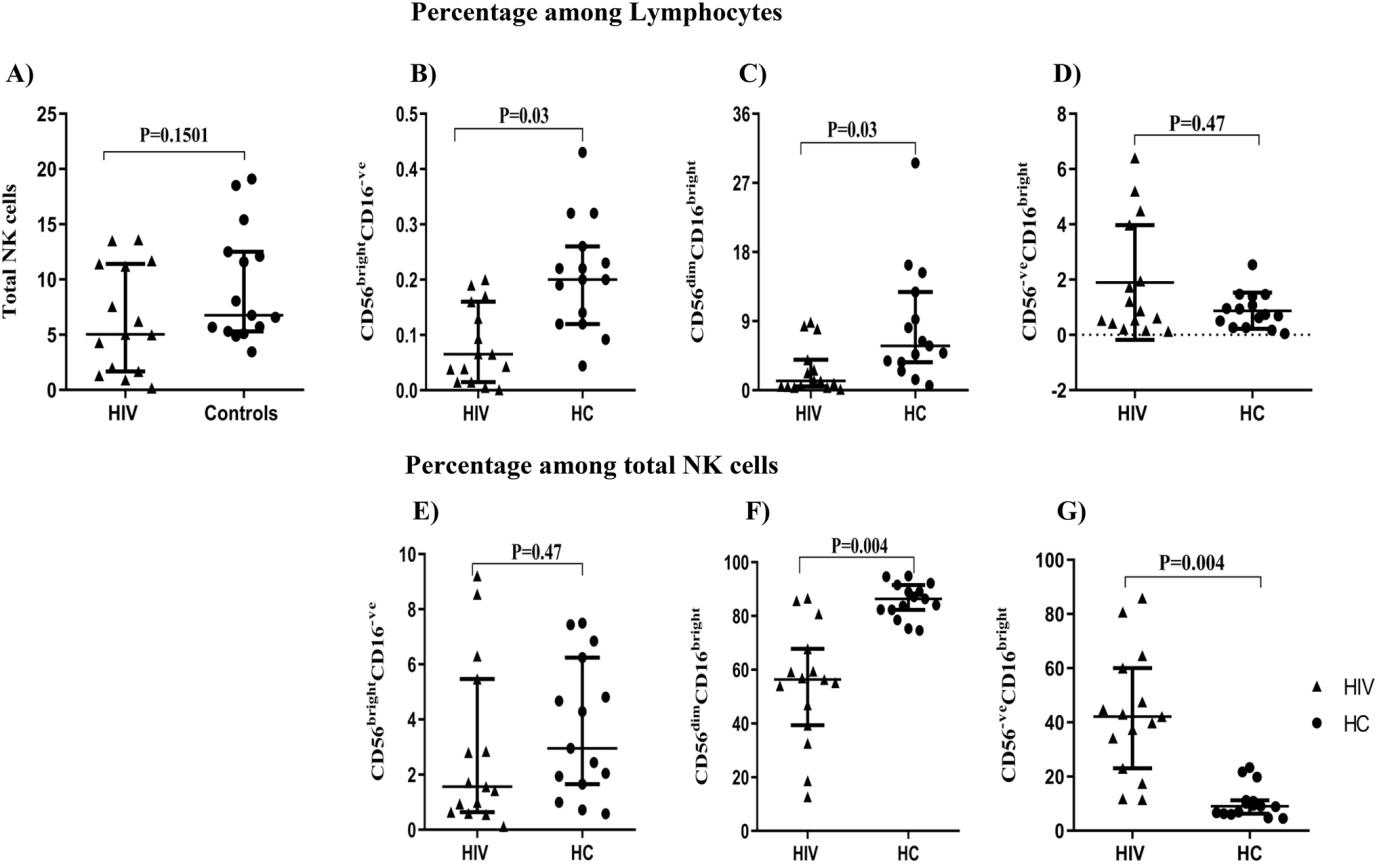
Total NK cells and subset distribution among the study groups. The frequency of total NK cells is indicated in the upper left corner, and was calculated based on the sum of CD56^bright^CD16^**−**ve^, CD56^dim^CD16^bright^, and CD56^**−**ve^CD16^bright^ subsets (**A**). The percentage of NK subsets was gated among lymphocytes, the great grandparent node as defined in Fig. 1 (**B**–**D**), or total NK cells, the sum of all three NK subsets (**E**–**G**) from HIV (triangles, n = 15) or HC (circles, n = 15). Mann–Whitney tests were performed with a p-value < 0.05 considered statistically significant. The median and 25th and 75th percentile are indicated on each plot.

**Figure 3:**
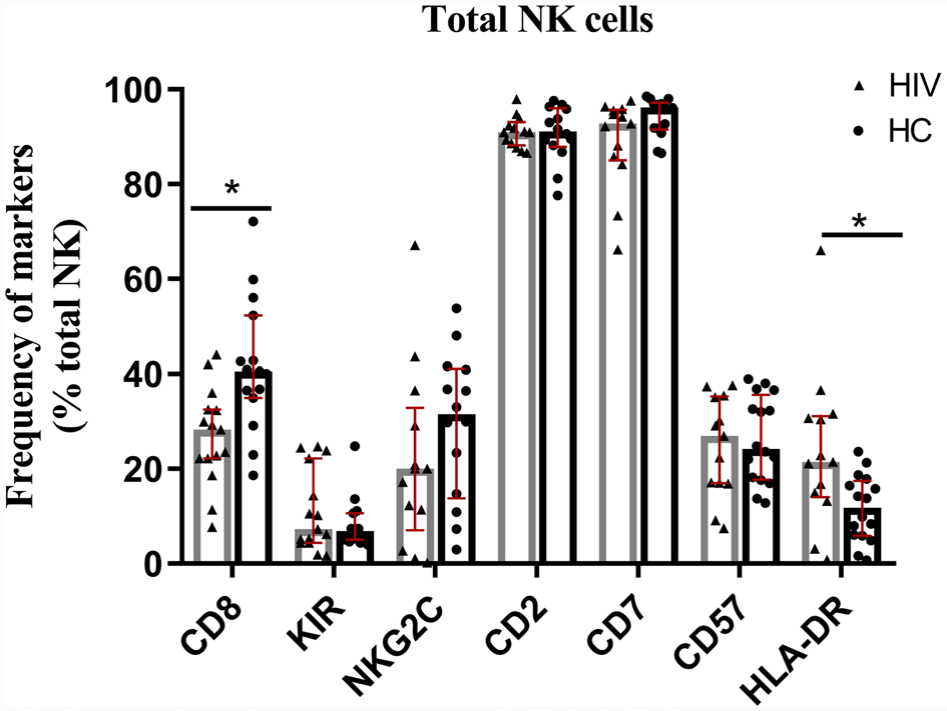
Expression of multiple markers on NK cells among the study groups. The analysis of CD8 and KIR3DL1/S1 expression was quantified on total NK cells comprising CD56^bright^CD16^**−**^, CD56^dim^CD16^bright^, and CD56^**−**ve^CD16^bright^, whereas the expression of NKG2C, CD2, CD7, CD57, and HLA-DR were determined from NK cells comprising the CD56^bright^ and CD56^dim^ subsets. A total of 15 subjects were evaluated for HIV and 15 for HC subjects for CD8 and KIR markers; 13 (HIV) and 14 (HC) subjects for NKG2C, CD2, CD7; and 13 (HIV) and 16 (HC) subjects for CD57 and HLA-DR marker expression. Abbreviations on X-axis refer to the different NK cell markers. Mann–Whitney tests were performed with a p-value < 0.05 considered statistically significant. The median and 25th and 75th percentile are indicated on each plot.

In this study, we defined NK cells by the expression of CD56 and/or CD16 among CD3 negative cells into three subsets: CD56^bright^CD16^−ve^, CD56^dim^CD16^bright^, and CD56^−ve^CD16^bright^ (Fig. 1). In some experiments, only CD56 was used to define CD56^bright^ and CD56^dim^ subsets, as described previously in the literature^22^. The frequency of total NK cells (Fig. 2) and the subsets (Fig. 3) were subsequently analyzed and compared across study groups.

### NK cell subset distribution among the study groups

The frequency of total NK cells and subsets thereof were analyzed and compared between HC and HIV groups. We assessed NK cell subsets as a fraction of total NK cells (defined as the sum of the three subsets) or as a fraction of total lymphocytes (Fig. 2). Among lymphocytes, we observed a lower frequency of both CD56^dim^CD16^bright^ and CD56^bright^CD16^−ve^ in HIV compared to HC subjects (p = 0.03 and 0.03, respectively). There was no significant difference in the CD56^−ve^CD16^bright^ NK cell subset frequency between the groups. However, when frequencies were analyzed among total NK cells, we continued to note a significantly lower frequency in the CD56^dim^CD16^bright^ subset (p = 0.004), but a significantly higher frequency in the CD56^−ve^CD16^bright^ subset in the HIV compared to HC groups (p = 0.004). Furthermore, among total NK cells, a strong inverse correlation was observed between CD56^−ve^CD16^bright^ and CD56^dim^CD16^bright^ subsets (r = − 0.9296, p = 0.0001).

### Expression of CD8, KIR, NKG2C, CD2, CD7, CD57, and HLA-DR on NK cells

We examined total NK cells for the expression of CD8, KIR3DL1/S1 (CD158e1/e2), CD159C (NKG2C), CD2, CD7, CD57, and HLA-DR between the healthy control (HC) and HIV groups. As shown in Fig. 3, NK cells from the HIV group had a significantly lower frequency of CD8^+^ cells (p = 0.001) and a higher frequency of HLA-DR^+^ cells (p = 0.045), relative to HC. In contrast, the expression of KIR3DL1/S1, NKG2C, CD7, and CD57 did not significantly differ between the cohorts.

### The expression of NK cell receptors within the subsets

**Figure 4:**
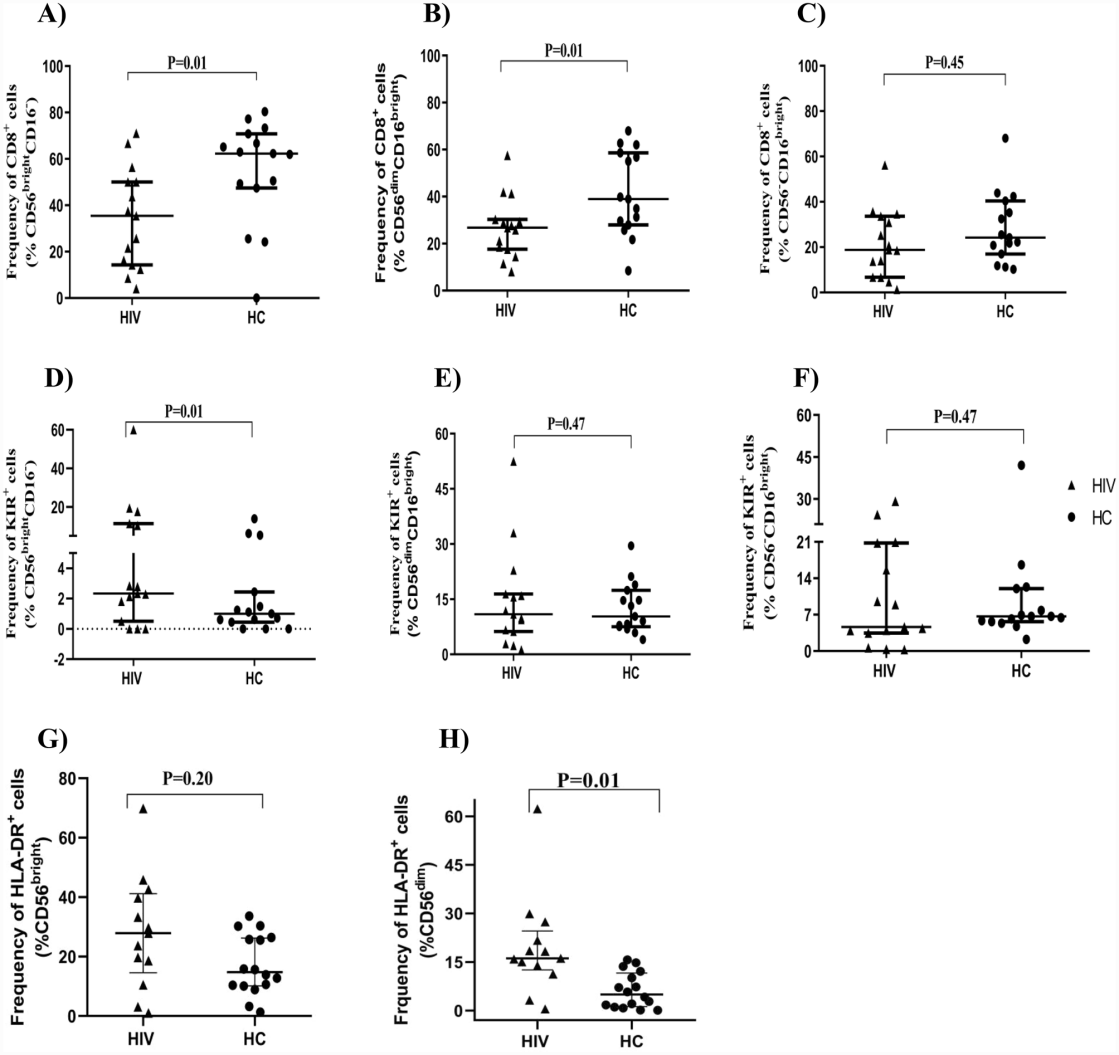
Frequency of KIR3DL1/S1^+^, CD8^+^ and HLA-DR cells among NK cells subsets. The frequency of CD8^+^ and KIR3DL1/S1^+^ cells within the CD56^bright^CD16^**−**ve^ (**A**,**D**), CD56^dim^CD16^bright^ (**B**,**E**) and CD56^**−**ve^CD16^bright^ (**C**,**F**) subsets are depicted for both HIV (n = 15) and HC groups (n = 15). The frequency of HLA-DR positive cells is shown for CD56^bright^ (**G**) and CD56^dim^ (**H**) subsets (n = 13, HIV group, n = 16 HC group). Mann–Whitney tests were performed with a p-value < 0.05 considered statistically significant. The median and 25th and 75th percentile are indicated on each plot.

To determine the differences in the expression of different NK cell markers, we first examined the frequencies of CD8 and CD158e1/e2 (KIR3DL1/S1) expressing cells among the three subsets. We found a small percentage of KIR^+^ cells among the CD56^bright^CD16^−ve^ subset, and this frequency was significantly higher (p = 0.01) in HIV than in HC subjects, shown in Fig. 4. Notably, KIR frequencies were higher in the other two NK subsets as well, though these differences did not reach statistical significance. The frequency of CD8^+^ cells was significantly lower in HIV compared with HC subjects within the CD56^bright^CD16^−ve^ (p = 0.01), and CD56^dim^CD16^bright^ (p = 0.01) subsets, whereas CD8 frequencies among the CD56^−ve^CD16^bright^ subsets were similar in the two subject cohorts (p = 0.47).

In addition, we evaluated the surface expression of CD57, HLA-DR, and NKG2C markers, focusing on the CD56^bright^ and CD56^dim^ NK subsets. As shown in Fig. 4, the frequency of HLA-DR^+^ cells on the CD56^dim^ subset was significantly higher in the HIV group than in HC (p = 0.01). We did not detect significant differences between the HIV and control cohorts in the expression of CD57 and NKG2C among CD56^bright^ and CD56^dim^ (data not shown).

### Expression of CD7 and CD2 by NK cell subsets

**Figure 5:**
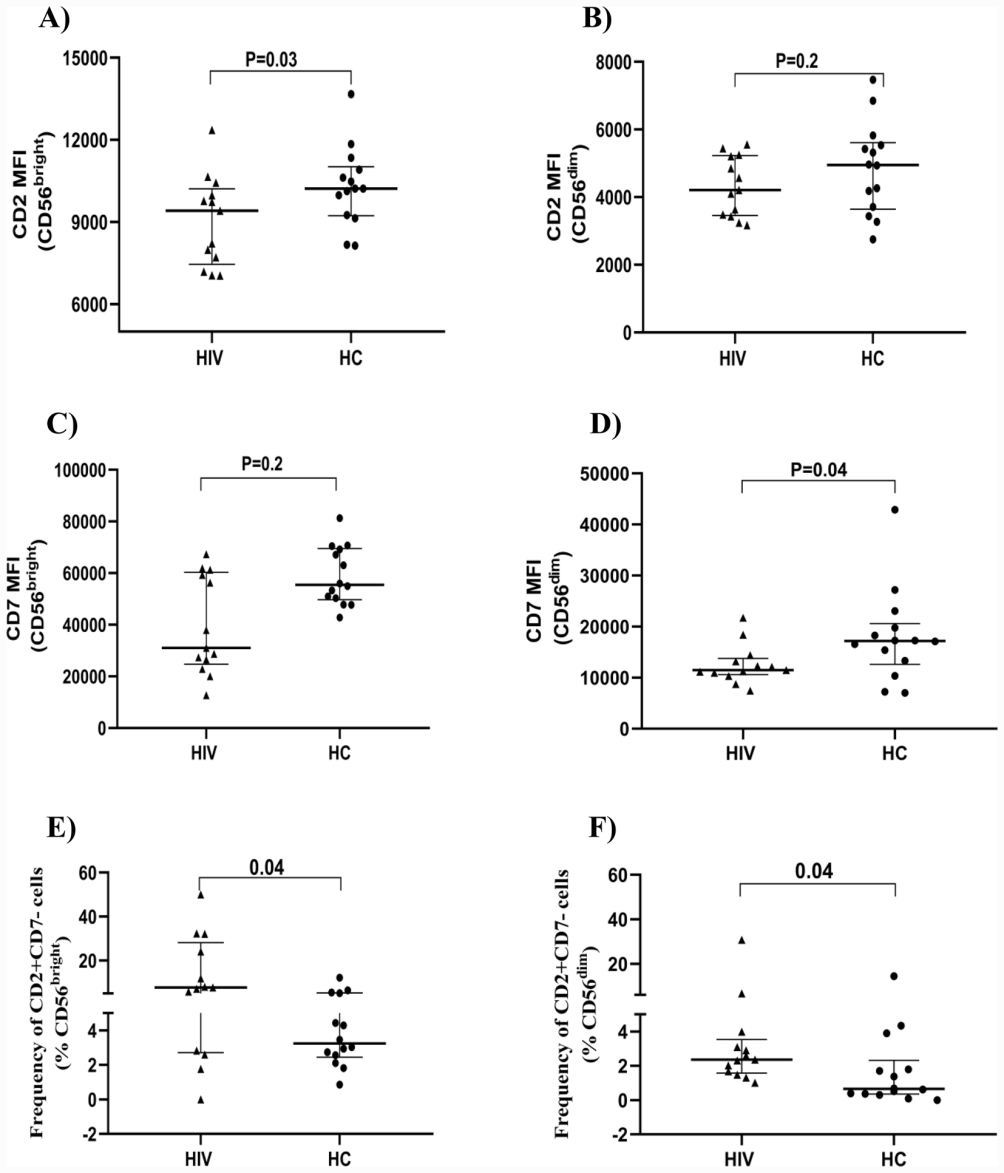
Expression of CD2 and CD7 by the subsets. The dot plot graph depicts the median fluorescent intensity of CD2 and CD7 on CD56^bright^ (**A**,**C**) and CD56^dim^ (**B**,**D**) NK cell subsets (HIV = 13, HC, 14 subjects). The frequency of CD7^−^CD2^+^ cells among CD56^bright^ (**E**) and CD56^dim^ (**F**) subsets is depicted. Mann–Whitney tests were performed with a p-value < 0.05 considered statistically significant. The median and 25th and 75th percentile are indicated on each plot.

We further evaluated the two subsets for the expression of CD2 and CD7 (Fig. 5). CD2 is a major coactivating receptor expressed on NK that recognizes CD58, a ligand expressed on a wide variety of tissues. Most NK cells express CD7, the density of which may be related to maturation or activation^11,23^. We found no significant differences in the frequency of CD7 expressing NK cells between the HIV and HC study groups. However, the density of CD7, as indicated by the median fluorescent intensity, was lower in HIV patients. This was apparent in total NK cells (p = 0.02). and among the CD56^dim^ subset the density was lower among HIV than HC (p = 0.04)). CD7 was also lower among HIV patients within the CD56^bright^ subset, but this did not reach statistical significance (p = 0.2).

The frequency of CD2 positive cells did not differ between the two cohorts among either total NK cells, or CD56^bright^ or CD56^dim^ subsets. Furthermore, we did not observe differences in CD2 density between HIV or HC subjects (p = 0.3). However, the CD56^bright^CD16^−ve^ subset did exhibit lower CD2 density among HIV relative to HC (p = 0.03). A significant difference between the two cohorts was not seen among CD56^dim^ cells (p = 0.2).

Co-expression of CD7 and CD2 was evaluated on both the CD56^bright^ and CD56^dim^ subsets. There was a large proportion of CD7^+^CD2^+^ cells within both subsets, without differences in the two clinical cohorts. However, we observed a significantly higher frequency of CD7^−^CD2^+^ cells among both CD56^bright^ (p = 0.04) and CD56^dim^ (p = 0.04) subsets (Fig. 5E,F).

### Correlation of NK cell subsets and molecules with viral load

We assessed whether any of the frequencies of NK subsets or molecules expressed on these subsets were associated with viral load. Neither NK cells, the three NK subsets, nor any of the molecules expressed on them showed statistically significant correlations with viral load. However, three populations did show borderline associations. NKG2C expressing NK cells were inversely correlated with viral load (r = − 0.47, p = 0.055). Similarly, CD57 NK cells were negatively associated with viral load (r = − 0.46, p = 0.08), whereas CD2^+^CD7^−^ NK cells were positive correlated with viral load (r = 0.53, p = 0.06).

## Discussion

NK cells represent an important component of the immune response and are associated with reduced HIV disease progression both in observational studies and in vaccine trials^24–26^. Since many of the studies on NK phenotype and function have been performed in developed countries and few in resource limited regions and given that local factors could impact disease, the goal of the current study was to characterize the phenotypic of NK subsets among HAART naïve HIV subjects compared with healthy controls in the Ethiopian setting. Our key findings are as follows: (1) The three previously identified NK subsets, CD56^bright^CD16^−ve^, CD56^dim^ CD16^bright^ and CD56^−ve^CD16^bright^, were observed to significantly change according to patterns previously identified when evaluated as a fraction of total NK cells. However, the CD56^−ve^CD16^bright^ subset, hypothesized to expand in HIV infection, was not found to be significantly changed when expressed as a function of total lymphocytes, raising the possibility that in our cohort much of the observed effect may not be related to expansion. (2) We identified elevated levels of HLA-DR molecules on NK, particularly the CD56^dim^CD16^bright^ subset confirming previous studies. Moreover, we observed down-regulation of CD7 molecules in these subsets, presumably a related effect of NK activation. (3) We confirmed other research illustrating a significant decrease in CD8 positive NK cells among HIV infected subjects. Finally, we observed a relatively high fraction of KIR3DL1 positive cells among the CD56^bright^CD16^−ve^ NK cells in HIV patients, an unexpected finding given current views of the CD56^bright^CD16^−ve^ as a relatively immature and KIR negative population.

The CD56^−ve^CD16^bright^ population has gained attention for its overexpression in HIV patients, with further studies illustrating reduced functional capacity in these cells. This has led to the conclusion that it represents an expanded population of NK cells. Initial evidence for this was obtained by demonstrating enhanced concentration of CD56^−ve^CD16^bright^ NK cells in the blood of HIV patients^9,11,27^. Many subsequent studies have assumed expansion based on the observed elevated percentage of this subset among total gated NK cells^28,29^. However, in this study, we observed significantly enhanced percentages of the CD56^−ve^CD16^bright^ cells only among gated NK cells, not among lymphocytes. This suggests an additional mechanism that may contribute to the apparent elevated frequency of CD56^−ve^CD16^bright^ cells, namely the depletion of other NK subsets. As in other recent studies, we did not assess NK subset number as a function of blood volume, but our findings suggest in the future this should be considered in parallel, rather than simply relying on the subset percentages of gated NK.

While it is clear that cell activation is crucial for protective immune cell function in general, and NK cell function in particular, it is well established that HIV pathogenesis is unusual in that widespread and inappropriate activation of the immune system occurs and can result in reduced function. In the context of NK cells, it is tempting to speculate that the elevated expression of HLA-DR molecules on NK cells in HIV patients reflects such a process. Indeed, some reports have indicated that frequencies of activated NK cells in HIV patients are predictive of a poor prognosis^14,22,30,31^. It is possible that such activation could be a prelude to the aforementioned differentiation into dysfunctional CD16^+^CD56^−ve^ cells^11^, enhanced susceptibilities to apoptosis leading to diminished NK numbers or reduced function by HIV products such as nef^32^. Given the role of translocated intestinal lumen bacterial products in immune activation in HIV patients^33^, it is also possible that such microbial products may contribute either directly or indirectly to NK activation via monocyte/dendritic lineage cells.

CD7 has been shown to be down-regulated as a consequence of activation on both NK cells and T cells. It may be seen at depressed levels in chronic diseases, with most evidence demonstrated in cancer models, and little evidence in HIV disease^34–36^. In the present study we observed reduced intensity of CD7 on NK cells in HIV patients. In addition, we also observed a small population of CD7^−^CD2^+^ cells enriched in these subjects. While CD7 negative cells have been reported, others have suggested these may represent myeloid lineage cells. Further studies will be required to clarify this, but given the generalized decrease in CD7 in HIV patients, it is conceivable that these could represent NK cells with down-regulated CD7 molecules. With the demonstrated functional importance of CD7^37,38^, our results suggest that further study of NK cells with reduced CD7 may shed further light on NK dysfunction in HIV disease.

We observed significantly depressed frequencies of CD8 expressing NK cells. This was observed equally among the CD56^dim^CD16^bright^ and CD56^bright^CD16^−ve^ subsets, confirming earlier studies that reported a selective loss of CD16^+^CD56^+^CD8^+^ or CD16^+^CD8^+^ NK cells in HIV infection^17,28,39^. Though not evaluated here, CD8 positive NK cells have been shown to exhibit polyfunctionality, with both high cytokine production and cytolytic capacities^17^, a property thought to be involved in protection by T cells against HIV progression.

KIRs are generally expressed by mature CD56^dim^CD16^bright^cells^40^. However, we observed a significant fraction of CD56^bright^CD16^−ve^ cells which were positive for KIR3DL1/S1. To our knowledge, only one other study reported such a finding in HIV patients^41^. While CD56^bright^CD16^−ve^ cells are relatively infrequent in blood they are more commonly expressed in tissues, and though typically KIR negative, increased KIR on decidual NK CD56^bright^CD16^−ve^ cells have been reported^42,43^. The relationship with CD56^bright^CD16^−ve^ cells in tissues with those in the blood is unclear^43^, but it is conceivable that under some conditions, a subset of CD56^bright^CD16^−ve^ cells may undergo differentiation expressing KIR and retaining a high level of CD56 at least during the initial stages. Such a possibility would also be consistent with an in vitro study in which CD56^bright^ NK cells activated in vitro retained the CD56^bright^ phenotype but acquired KIR over several days in culture^44^. Further studies are needed to address the role of KIR on CD56^bright^CD16^−ve^ cells in HIV disease.

Our study was limited by a relatively small sample size, reducing the power of the study. Several observations, in particular correlations of marker expression with HIV viral load, showed suggestive associations with borderline statistical significance. Future studies in this setting with larger sample sizes will be needed to confirm and extend these observations.

## Conclusions

This is the first study to comprehensively examine the phenotypes of multiple NK subsets in Ethiopia among HIV patients. We confirmed many of the previously defined phenotypic characteristics of NK cells described in multiple studies elsewhere. Further, our results highlight the importance of gating for enumeration of the overexpressed CD56^−ve^CD16^bright^ NK subset, the potential importance of CD7 as activation and functional marker for NK cells, and finally the overexpression of KIR3DL1/S1 among the CD56^bright^CD16^−ve^ subset among HIV patients, observations which have received little attention in previous studies.

## Materials and methods

### Study participants

A total of 31 study participants, 15 HAART naïve HIV patients (HIV group) and 16 healthy controls (HC) aged 18–49 years were enrolled in the study. In some experiments, a subset of these was utilized. The participants were recruited from health centers and hospitals in Addis Ababa, Ethiopia. All participants were screened for HIV by current national test algorithms and initial positives were further tested with the recency test (Asante recency test), with all patients exhibiting chronic disease > 6 months. HIV subjects were tested for viral load at it is a routinely done test, instead of CD4 T cell count. Healthy study participants were appointed to come back after a month, at which point the HIV test was repeated to confirm negativity. None of the participants had conditions such as tuberculosis, diabetes mellitus, pregnancy or were on treatment with immune suppressive medication (Supplementary Table S1).

### Staining of cell surface antigens and flow cytometric acquisition

Whole blood samples were collected from each participant by venous puncture, and whole blood staining was performed. Briefly, 100 μl of blood was transferred to each tube containing a mixture of antibodies labeled as: panel-1 with CD3-APC-Cy7 (Becton-Dickinson (BD), USA), CD16-FITC (BD, USA), CD56-PE (BD, USA), KIR3DL1/S1-APC (Beckman coulter), and CD8-PerCP (BD, USA), panel-2 with CD3-APC-Cy7, CD56-PE and NKG2C-Alexa488 (R&D, England), panel-3 with CD3-APC-cy7, CD56-PE, CD2-FITIC (Becton-Dickinson, USA) and CD7-PerCP (BD, USA), panel-4 with CD3-APC-cy7, CD56-PE, CD57-FITC (BD, USA), HLA-DR-PERCP (BD, USA) and CD19-APC (BD, USA), panel-5 with no antibody (unstained tube). The tubes were then incubated for 20 min in the dark at 4 °C. Red blood cells (RBCs) were lysed with 1 ml of BD FACS lysing solution for 10 min at room temperature, and washed with addition of 3 ml of FACS buffer (phosphate buffer saline 0.1 mM EDTA and 0.1% fetal bovine serum), centrifugation at 400×*g* for 7 min, and resuspended with 400 µl of FACS buffer. Compensation bead containing tubes were prepared with each fluorochrome and used during flow cytometer set up. 100,000 events were acquired from all tubes except panel-5 (20,000 events only), using a FACS Canto II flow cytometer equipped with FACSDiva software. In some experiments for some samples staining was poor resulting in sample exclusion from analysis, resulting in sample numbers for a given marker set less than the maximum 15 HIV and 16 HC subjects. Sample numbers are indicated in the figure legends.

### FlowJo analysis

FlowJo (BD) software was used for analysis with prepared templates, incorporating gates adjusted for each sample. Lymphocytes were gated based on forward scatter (FSC) and side scatter (SSC) and singlets were subsequently selected using an FSC-height (FSC-H) versus FSC-area (SSC-A) plot. After gating on CD3 negative cells, NK cells were then identified by the expression CD16 and/or CD56 as depicted in Fig. 1. Percentages of individual NK subsets (CD56^bright^CD16^−ve^, CD56^dim^CD16^bright^ and CD56^−ve^CD16^bright^) were defined among total NK cells defined as the sum of the three subsets. NK subsets were defined also as a percentage of lymphocytes as the great-grandparent node using FlowJo software. This figure is mathematically identical to 100 times the product of the fraction of singlets among gated lymphocytes, the fraction of CD3^−^ cells among gated singlets, and the fraction of the NK subset of interest. The three NK subsets were further analyzed for expression of CD8 and KIR3DL1/S1 using panel-1. However, for the rest of the panels, only CD56^dim^ and CD56^bright^ subsets were identified as depicted in Fig. 1 and analyzed for NKG2C, CD8, CD7, CD2, CD57, and HLA-DR**.**

### Data analysis

Data obtained from FlowJo Version 10.1 software and the viral load data from the Abbott machine were exported into JMP software (SAS, Cary, NC) after importation of an intermediate Excel (Microsoft) file for statistical analysis. Cell populations of interest were compared according to the study cohort as independent variables using the Mann–Whitney *U* test with p-values < 0.05 considered statistically significant. Spearman’s correlation was done to examine the correlation between NK subset frequencies and HIV viral load for HIV-infected participants.

### Ethical approval and informed consent

Participation in this study was voluntary and the study was approved by both the ethical review board at the University of Gondar, School of Biomedical and Laboratory science and Armauer Hansen Research Institute All Africa Leprosy, Tuberculosis, and Rehabilitation Training Center Ethics Review Committee (AERC), protocol number P006/18. Written informed consent was obtained from the study participants before involvement in the study. The AERC approved the study of the patient material collected. All experiments were performed in accordance with relevant guidelines and regulations.

## Supplementary Information

Supplementary Table S1.

## Data Availability

The datasets used and/or analyzed during the current study are available from the corresponding author on reasonable request.

## Abbreviations

CD: Cluster of differentiation
CLR: C-type lecithin receptor
DC: Dendritic cells
FACS: Fluorescent activated cell sorter
FSC: Forward scatter
HAART: Highly active anti-retroviral treatment
HIV: Human immune deficiency virus
HLA: Human leukocyte antigens
Ig: Immunoglobulin
KIR: Killer like immunoglobulin receptor
NK: Natural killer cells
SSC: Side scatter
AIDS: Acquired immune deficiency syndrome
N/A: Not applicable
