# Supplementary material for: Elevated KIR expression and diminished intensity of CD7 on NK cell subsets among treatment naïve HIV infected Ethiopians
Source: Sci Rep. 2022 Aug 30;12:14747. doi: 10.1038/s41598-022-18413-3 (PMC9427747; doi:10.1038/s41598-022-18413-3)
Supplement: Supplementary file 1 — Supplementary Table S1. [file 41598_2022_18413_MOESM1_ESM.pdf]

Elevated KIR expression and diminished intensity of CD7 on NK cell subsets among treatment naïve HIV infected Ethiopians

Henok Andualem[1*], Mulualem Lemma[2], Amare Keflie[2], Meseret Workeneh (MW)[2], Birhanu Ayelign[2], Yayehyirad Tassachew[3], Lidya Hailu[1], Alene Geteneh[4], Adane Mihret[5], Martha Zewdie[5], Rawleigh Howe[5]

[1] Department of medical laboratory science, college of medicine and health science, Debre Tabor University, Debre Tabor, Ethiopia.[2]Department of Immunology and molecular biology, school of medical laboratory science, college of medicine and health science, University of Gondar, Gondar, Ethiopia; [3] School of Medical Laboratory Sciences, College of Medicine and Health Sciences, Hawassa University, Hawassa, Ethiopia. [4] College of health science, Woldia University, Wondia, Ethiopia. [5]Armauer Hansen Research Institute (AHRI), Addis Ababa, Ethiopia.

Supplementary Table S1: The viral load on HAART naïve HIV patients

Patient ID	Viral Load<br><br>Copies/ml
**AL-002**	100122
**KOL-001**	275615
**PA-001**	41074
**AL-004**	209435
**AD-001**	166090
**AK-001**	35000
**AK-002**	5153
**KOL_002**	36775
**KOL-003**	173853
**AB-001**	105011
**AL-005**	98076
**KOL-004**	144895
**KOL-005**	4039
**AL-004**	209435
**KOL_002**	36775
